# Supplementary material for: Complexome profiling of the Chlamydomonas psb28 mutant reveals TEF5 as an early PSII assembly factor
Source: Plant Cell. 2025 Mar 17;37(6):koaf055. doi: 10.1093/plcell/koaf055 (PMC12164590; doi:10.1093/plcell/koaf055)
Supplement: koaf055_Supplementary_Data [file koaf055_supplementary_data.zip › psb28_interactive.zip!/plots/intensity/qualityPlots/peptides/ALD8_Cre16.g675650.t1.2.html]

### Cre16.g675650.t1.2

Trivial name: ALD8  
SeqCoverage [median]: 43.60  
Mass: 58.16 kDa  
  
 

| Sequence | MissedCleavage | Mass [Da] | wtIntensity | muIntensity | Score |
| --- | --- | --- | --- | --- | --- |
| AASDAFPK | 0 | 805.40 | 13151.17 | 26766.69 | 120.49 |
| AGVQFYTQTK | 0 | 1141.58 | 31325.98 | 42044.38 | 209.73 |
| AISFVGSDSAGK | 0 | 1137.57 | 25014.35 | 33120.76 | 155.11 |
| AISFVGSDSAGKYIYAR | 1 | 1803.92 | 0.00 | 1930.78 | 30.13 |
| ALAGAAFGAAGQR | 0 | 1159.61 | 17261.72 | 31914.05 | 221.16 |
| ANMEELAR | 0 | 932.44 | 16958.54 | 33442.48 | 156.01 |
| DADVGPMISPEAK | 0 | 1328.63 | 1932.03 | 7700.91 | 150.46 |
| DPGAAVMLADLAQQAGLPK | 0 | 1864.97 | 8034.56 | 11674.22 | 204.13 |
| FQELIR | 0 | 804.45 | 5492.27 | 13812.09 | 92.32 |
| GISAPAATTSSSSGSK | 0 | 1407.68 | 27499.22 | 38293.83 | 236.08 |
| GLKLNAGWEKDADVGPMISPEAK | 2 | 2425.23 | 0.00 | 2130.76 | 46.18 |
| GSFAGDLHMYGR | 0 | 1309.59 | 16121.68 | 25560.74 | 97.91 |
| GVLNIVQGSR | 0 | 1041.59 | 33310.93 | 101556.00 | 214.96 |
| GVSVPGYER | 0 | 962.48 | 13215.36 | 19438.89 | 101.33 |
| LIASGAAAGAQVLLDGR | 0 | 1581.88 | 11977.94 | 47855.52 | 233.52 |
| LNAGWEKDADVGPMISPEAK | 1 | 2127.03 | 4520.41 | 9553.74 | 109.72 |
| NHAVVMPDADVDSTVK | 0 | 1696.81 | 26258.26 | 37465.85 | 116.58 |
| RVQANLGAK | 1 | 955.56 | 1237.48 | 767.84 | 75.11 |
| SEFNAAVK | 0 | 864.43 | 13906.45 | 37713.49 | 161.90 |
| SVTMEQGK | 0 | 878.42 | 836.26 | 4956.98 | 113.50 |
| SVTMEQGKTLADAR | 1 | 1505.75 | 0.00 | 1367.70 | 95.66 |
| TVTAKWPAEDIR | 1 | 1385.73 | 0.00 | 710.99 | 45.26 |
| VMFKFQELIR | 1 | 1309.72 | 0.00 | 2287.43 | 53.68 |
| VQANLGAK | 0 | 799.46 | 14237.00 | 37077.03 | 125.01 |
| WREPLLEAAR | 1 | 1239.67 | 0.00 | 443.22 | 33.09 |
|  |  |  | 282291.6 | 569586.4 |  |
